# ^29^Si{^27^Al}, ^27^Al{^29^Si} and ^27^Al{^1^H} double-resonance NMR spectroscopy study of cementitious sodium aluminosilicate gels (geopolymers) and gel–zeolite composites

**DOI:** 10.1039/c8ra09246j

**Published:** 2018-12-07

**Authors:** Sebastian Greiser, Gregor J. G. Gluth, Patrick Sturm, Christian Jäger

**Affiliations:** Division 1.3 Structure Analysis, Bundesanstalt für Materialforschung und -prüfung (BAM) Richard-Willstätter-Str. 11 12489 Berlin Germany christian.jaeger@bam.de; Division 7.4 Technology of Construction Materials, Bundesanstalt für Materialforschung und -prüfung (BAM) Unter den Eichen 87 12205 Berlin Germany gregor.gluth@bam.de

## Abstract

The influence of starting materials and synthesis route on the properties and the structure of cementitious sodium aluminosilicate gels is not fully understood, partly due their amorphous nature and the fact that they often contain residual reactants, which can make the results of single-pulse NMR spectroscopy applied to these materials difficult to interpret or ambiguous. To overcome some of these limitations, ^29^Si{^27^Al} TRAPDOR NMR as well as ^27^Al{^29^Si} and ^27^Al{^1^H} REDOR NMR spectroscopy were applied to materials synthesized by the one-part alkali-activation route from three different amorphous silica starting materials, including rice husk ash. The latter led to formation of a fully amorphous sodium aluminosilicate gel (geopolymer), while the materials produced from the other silicas contained amorphous phase and crystalline zeolites. Application of the double-resonance NMR methods allowed to identify hydrous alumina gel domains in the rice husk ash-based material as well as significantly differing amounts of residual silica in the three cured materials. Four-coordinated Al existed not only in the aluminosilicate gel framework but also in a water-rich chemical environment with only a small amount of Si in proximity, likely in the alumina gel or possibly present as extra-framework Al in the aluminosilicate gel. The results demonstrate how the employment of different silica starting materials determines the phase assemblage of one-part alkali-activated materials, which in turn influences their engineering properties such as the resistance against chemically/biologically aggressive media.

## Abstract 2

Double-resonance NMR spectroscopy applied to one-part alkali-activated materials shows how the silica starting material determines their phase assemblage.

## Graphical abstract

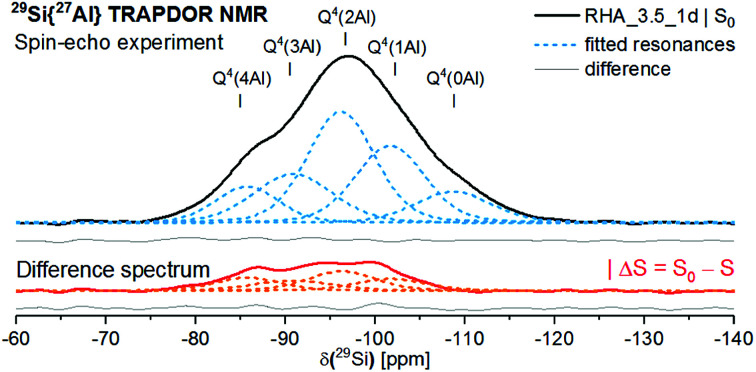

## 1 Introduction

Cementitious sodium aluminosilicate gels (sometimes referred to as aluminosilicate inorganic polymers or geopolymers) can be produced by activation of sufficiently reactive low-calcium (alumino)silicate precursors, such as metakaolin, fly ash or volcanic rock, with alkaline solutions, such as alkali hydroxide or alkali silicate solutions.^1^ These materials possess beneficial engineering properties in a range of ‘cement-like’ applications, *i.e*. as binders for mortars and concretes, particularly for applications where high resistance against chemical attack^2–5^ or against high temperatures^6–8^ is required. In addition, they possess characteristics that make them promising candidates for use as fire-proofing and refractory materials.^9–12^ Cementitious sodium aluminosilicate gels are also extensively studied with regard to radioactive waste stabilization^13^ and other applications that make use of their sorption properties.^14,15^ However, despite intensive research regarding these various applications, their nanostructure and how it influences their properties (*e.g.*, their durability in cement-like applications^16,17^) is not fully understood.

The current knowledge of the nanostructure of these sodium aluminosilicate gels and the influence of their chemical composition on the former is based mainly on ^29^Si single-pulse MAS NMR studies, complemented by ^27^Al and ^23^Na single-pulse MAS NMR data.^18–23^ Additional information, in particular on the state of water in these gels, has been obtained by means of ^1^H MAS NMR, ^1^H–^29^Si CPMAS NMR as well as two-dimensional and multiple-resonance NMR methods.^23–26^ The structure that emerges from these studies is an amorphous framework silicate with partial substitution of Al^3+^ for Si^4+^ in tetrahedral sites. Si in these gels is present in Q^4^(*m*Al) sites with *m* = 1, 2, 3, and 4, the relative amounts of which depend on its overall SiO_2_/Al_2_O_3_ ratio. Surface –OH groups occur only to a very limited extent in these gels. The negative framework charge caused by the Al^3+^–Si^4+^ substitution is thought to be balanced by Na^+^ ions and possibly by extra-framework Al (EFAL); the results of different groups differ regarding the locations and the state of hydration of the charge balancing Na^+^ ions. Because of the above characteristics, cementitious sodium aluminosilicate gels are regarded as being closely related to the amorphous precursors of zeolite synthesis (and thus also related to crystalline zeolites to some extent),^27^ albeit produced under water-deficient conditions.

Very recently, a refined structural model has been put forward based on ^27^Al, ^23^Na, and ^17^O 3QMAS NMR,^28^ in which the charge balancing species are Na^+^ ions coordinated to three framework oxygen atoms and three H_2_O molecules, Na^+^ ions coordinated to four framework oxygen atoms and two H_2_O molecules, and six-coordinated EFAL. Differing from this proposal, an earlier study^25^ that employed ^27^Al 3QMAS and ^27^Al{^1^H} REDOR 3QMAS NMR concluded that charge-balancing EFAL in sodium aluminosilicate gels is in four-fold coordination. Only in one case,^29^ an attempt was made to examine the structural model in terms of Si–O–Al connectivity by means of a ^29^Si{^27^Al} double-resonance NMR method. In that study, ^29^Si{^27^Al} REAPDOR NMR was employed to confirm the assignment of the resonances in the ^29^Si single-pulse MAS NMR spectrum of a metakaolin-based sodium aluminosilicate gel to the different Q^4^(*m*Al) units, though these units were already well separated in the single-pulse spectrum.

In the present study, ^29^Si{^27^Al} TRAPDOR NMR, ^27^Al{^29^Si} REDOR NMR and ^27^Al{^1^H} REDOR NMR were used to facilitate accurate structural description of cementitious sodium aluminosilicate gels, produced by alkali-activation of rice husk ash *via* the so called ‘one-part’ synthesis route.^30^ For comparative purposes, sodium aluminosilicate gel–zeolite composites produced by alkali-activation of other silica materials were studied too. It is demonstrated that the use of double-resonance NMR methods provides new insights into the phase assemblage and structure of these materials that is otherwise difficult to obtain. The obtained results highlight that the knowledge about conventional alkali-activated materials cannot be simply transferred to one-part alkali-activated cements without modifications, and in addition point to a convenient way to tune the properties of the latter materials.

## 2 Experimental

### 2.1 Materials

The starting materials for the synthesis of the sodium aluminosilicate gel and the sodium aluminosilicate gel–zeolite composites (in the following referred to as ‘composites’) were sodium aluminate, a rice husk ash (denoted ‘RHA’), a commercial microsilica (denoted ‘MS’) and a silica-rich by-product from chlorosilane production (denoted ‘CR’). The chemical compositions of the starting materials are shown in Table 1. Except the sodium aluminate, all starting materials were almost completely amorphous, as determined by powder X-ray diffraction (XRD), and they had comparable specific surface areas in the range 20–50 m^2^ g^−1^. The sodium aluminate consisted almost completely of NaAlO_2_ and contained minor amounts of NaAlO_2_·1.25H_2_O and natrite (Na_2_CO_3_) as impurities; it had a median particle size of 17.3 μm, as determined by laser granulometry. A more detailed description of the starting materials has been provided elsewhere.^31,32^

**Table 1:** Chemical compositions of the starting materials (in wt%)^a^

Component	Sodium aluminate	MS	CR	RHA
SiO_2_	0.61	95.16	84.23	88.49
Al_2_O_3_	60.85	0.17	4.18	0.58
Fe_2_O_3_	0.06	0.04	0.43	0.31
TiO_2_	n.d.	<0.01	0.06	0.03
CaO	0.26	1.71	2.97	1.00
MgO	0.02	0.28	0.17	0.88
Na_2_O	36.07	0.19	0.22	0.24
K_2_O	0.03	0.65	0.03	2.91
SO_3_	0.16	0.25	0.16	0.54
Cl^−^	n.d.	n.d.	1.36	n.d.
P_2_O_5_	n.d.	n.d.	n.d.	1.83
LOI	1.73	1.12	5.08	2.48

a n.d. not determined, LOI loss on ignition at 1000 °C.

Pastes were obtained by first dry-mixing the sodium aluminate with RHA, MS or CR in relative amounts to obtain a molar SiO_2_/Al_2_O_3_ ratio of the mixture of ∼3.5 (*i.e*. Si/Al ≈ 1.75) and subsequent mixing with water at a nominal water/solids ratio of 0.50. The resulting molar ratios of the pastes are shown in Table 2; sample designations refer to the silica starting material, the approximate SiO_2_/Al_2_O_3_ ratio and the curing time of 1 day, as described in the following. The pastes were mixed in a planetary centrifugal mixer either for 3 min at 1750 rpm (MS_3.5_1d and CR_3.5_1d) or for 4 min at 1200 rpm (RHA_3.5_1d). Subsequently, the pastes were cast into cubic (20 mm × 20 mm × 20 mm) molds and cured in the open molds at 80 °C and a relative humidity of ≥80% for 1 day in an oven with humidity conditioning. After this curing time, the hardened specimens were removed from the oven and the molds, allowed to cool down to room temperature and subsequently either tested for unconfined compressive strength or ground manually with mortar and pestle (agate) for further analyses. The compressive strengths were 17.7 MPa for MS_3.5_1d; 9.9 MPa for CR_3.5_1d; and 29.8 MPa for RHA_3.5_1d. The powdered samples for NMR analyses were stored in closed glass vials at laboratory temperature until required for testing.

**Table 2:** Molar oxide ratios of the starting mixtures

Material	Na_2_O/Al_2_O_3_	SiO_2_/Al_2_O_3_	H_2_O/Na_2_O
MS_3.5_1d	0.98	3.38	11.07
CR_3.5_1d	0.89	3.13	11.92
RHA_3.5_1d	1.00	3.48	11.85

As reported previously,^26,31^ MS_3.5_1d and CR_3.5_1d yielded composites containing mainly zeolite Na-A [Na_4_(AlSiO_4_)_4_·9H_2_O] and an amorphous sodium aluminosilicate gel with a molar SiO_2_/Al_2_O_3_ ratio of ∼2 (Si/Al ≈ 1). In the case of MS_3.5_1d, some hydrosodalite [Na_6_(AlSiO_4_)_6_·4H_2_O] was present in addition, while for CR_3.5_1d very minor amounts of a faujasite-type zeolite and/or zeolite Na-EMT were observed by XRD. The degree of reaction of the silica starting materials (MS and CR, respectively) in these composites was approx. 55–60%, meaning that the composites contained undissolved silica particles too; the latter were found to have a partially leached, hydrated surface layer. For RHA_3.5_1d, it was found by XRD that the reaction products were completely amorphous, except minor amounts of thermonatrite (Na_2_CO_3_·H_2_O) that had formed due to slight carbonation of the alkaline paste.^32^ In addition, the XRD patterns indicated that the degree of reaction of the silica starting material of RHA_3.5_1d was substantially higher than the degree of reaction of the starting materials of the composites (MS and CR).^32^

### 2.2 NMR experiments

To obtain information about the chemical environment of Si and Al in the studied materials, ^29^Si{^27^Al} TRAPDOR NMR,^33,34^^27^Al{^29^Si} REDOR NMR and ^27^Al{^1^H} REDOR NMR^35,36^ experiments were performed. These experiments have in common that first a spin-echo experiment is performed on the nucleus under study (spectrum denoted *S*_0_). Subsequently, the spin-echo experiment is repeated while a continuous wave radiofrequency (r.f.) pulse (TRAPDOR) or distinct 180° pulses (REDOR) are applied to a second nucleus whose influence is to be investigated. This leads to attenuation of the intensity of the signal of the nucleus under study, depending on the spin-echo times and particularly for those sites which have the second nucleus in spatial proximity (spectrum denoted *S*). In the difference of the latter two spectra, Δ*S* = *S*_0_ − *S*, the sites with the second nucleus in proximity are emphasized. The ratio Δ*S*/*S*_0_ is denoted TRAPDOR fraction or REDOR effect, respectively, and is a measure of the influence (*i.e*. abundance and proximity) of the second nucleus on the nucleus under study. The magnitude of the TRADPOR fraction or REDOR effect for a specific site depends, however, not only on the abundance/proximity of the second nucleus but also on other structural parameters, such as the overall Si/Al ratio in the case of aluminosilicates.^37^

^29^Si{^27^Al} TRAPDOR NMR experiments were performed on a Bruker DMX 400 spectrometer at 9.4 T, using a 7 mm triple-resonance probe and employing a sample spinning speed of 6.0 kHz. The ^29^Si 90° and 180° pulse lengths were 9.8 μs and 19.6 μs, respectively. A 15° two-phase pulse modulation (TPPM) sequence^38^ was applied for proton decoupling. The r.f. field strength of the ^27^Al TRAPDOR pulse was 25 kHz, corresponding to the selective ^27^Al 90° pulse length of 10 μs for YAG's AlO_4_ peak. Approximately weekly, measurements of the TRAPDOR pulse power were taken directly at the power amplifier with a 60 dB attenuator and an oscilloscope; the measured pulse power was always in the range 125 ± 3 W. An echo time of 2*τ* = *NT*_r_ = 52*T*_r_ = 8.6667 ms (*N*: number of rotor revolutions; *T*_r_: rotor period) was applied. 4672 scans with a recycle delay of 90 s were accumulated for each specimen. Thermal effects that could differently affect the two parts of the experiment if no measures are taken, where excluded by alternating measurements with and without ^27^Al TRAPDOR irradiation.^37^

For RHA_3.5_1d, a ^29^Si single-pulse MAS NMR spectrum was recorded on the same spectrometer. A sample spinning speed of 6.5 kHz and a 90° pulse of 7.5 μs duration were employed. The 15° TPPM sequence was applied for proton decoupling, and 128 scans with a recycle delay of 1200 s were accumulated. The recycle delay was chosen to ensure a quantitative measurement (recycle delay of at least five times the longest relaxation time, *T*_1_, of all components)^39^ despite the comparatively long *T*_1_ that may occur for some species in the present samples.^31^

^27^Al{^29^Si} REDOR NMR and ^27^Al{^1^H} REDOR NMR experiments were performed on a Bruker AVANCE 600 spectrometer, using a 4 mm triple-resonance probe and employing a sample spinning speed of 12.5 kHz. The ^27^Al 90° and 180° pulse lengths were 2.2 μs and 4.4 μs, respectively, previously determined for the AlO_4_ peak of YAG. The 15° TPPM sequence was applied for proton decoupling. The 180° REDOR pulse lengths were 7 μs for ^1^H and 14 μs for ^29^Si. 11 different echo times, 2*τ* = *NT*_r_ = 0.16, 0.32, 0.48, 0.80, 1.28, 2.40, 4.00, 5.60, 8.00, 12.00 and 16.00 ms, were applied in both experiments. 1024 and 4096 scans were accumulated for each specimen in the ^27^Al{^1^H} and the ^27^Al{^29^Si} REDOR NMR experiments, respectively. Before and after each ^27^Al{^29^Si} REDOR NMR experiment on a sample, *S*_0_ and *S* spectra were obtained on kaolinite for dephasing pulse lengths of 11, 12, 13, 14 and 15 μs, accumulating 256 scans for each spectrum. The minimum *S* signal was always obtained for the chosen (14 μs) pulse length, verifying the stability of the spectrometer and optimum choice of the experimental parameters.

All ^29^Si NMR spectra were referenced to kaolinite with its upfield resonance at −91.5 ppm, and all ^27^Al NMR spectra were referenced to YAG with its AlO_6_ resonance at 0.6 ppm. Deconvolution/fitting of the spectra was performed with the SOLA module of the Bruker TopSpin software, version 3.1. To facilitate unbiased fitting of the double-resonance spectra, the *S*_0_ spectrum and the *S* spectra for each echo time were summed for each sample, and the resulting sum spectrum was evaluated to obtain the chemical shift, the FWHM and the line shape (composed of Gaussian and Lorentzian) of the deconvoluted peaks. These parameters were then fixed to obtain the intensities of the resonances separately for the *S*_0_ spectrum and the *S* spectra for each echo time, respectively. To obtain the intensities of the resonances in the ^29^Si single-pulse MAS NMR spectrum of RHA_3.5_1d, the chemical shifts, FWHMs and line shapes determined for its ^29^Si{^27^Al} TRAPDOR NMR spectrum were adopted for the fit of the single-pulse spectrum. The Q^*n*^(*m*Al) nomenclature for SiO_4_ tetrahedra is used throughout this article, where *n* denotes the number of oxygen-bridges to neighboring SiO_4_ and AlO_4_ tetrahedra, and *m* ≤ *n* denotes the number of AlO_4_ of these tetrahedra; for *m* = 0, the expression in parentheses is omitted.

## 3 Results and discussion

### 3.1 ^29^Si{^27^Al} TRAPDOR NMR

**Fig. 1:**
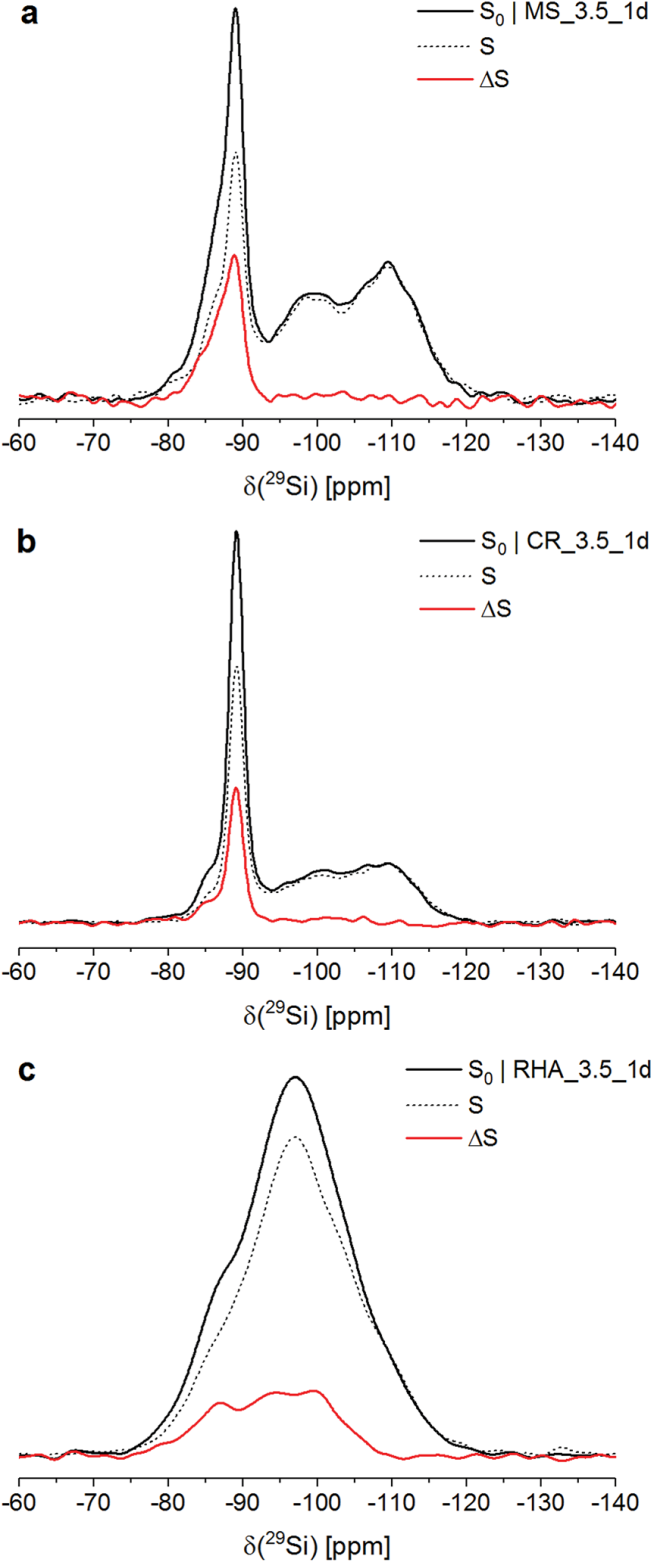
^29^Si{^27^Al} TRAPDOR NMR spectra of MS_3.5_1d (a), CR_3.5_1d (b) and RHA_3.5_1d (c). *S*_0_ (full black lines): spectrum obtained without ^27^Al TRAPDOR pulse; *S* (dashed black lines): spectrum obtained for an echo time of 8.6667 ms (*N* = 52); Δ*S* (full red lines): difference spectrum Δ*S* = *S*_0_ − *S*.

Fig. 1 shows the ^29^Si{^27^Al} TRAPDOR spectra of the three materials. The *S*_0_ spectra of all materials were very similar to their respective ^29^Si single-pulse MAS NMR spectra (not shown; for MS_3.5_1d and CR_3.5_1d, see ref. 26 and 31); thus, the Si speciation in these materials can be discussed on the basis of the former.

In the *S*_0_ spectra of MS_3.5_1d and CR_3.5_1d, the broad resonances at approx. −110 ppm and −98 ppm are assigned to Q^4^ sites in unreacted silica and Q^3^ sites in its partially hydrated (leached) surface layer, respectively. The major peak with its maximum at approx. −89 ppm and its downfield shoulder, present in both composites, are attributed to Q^4^(4Al) sites in three different phases: The peak at −89 ppm is assigned to zeolite Na-A. Sample MS_3.5_1d contained hydrosodalite, which causes a resonance at −87 ppm, visible as a shoulder in its *S*_0_ spectrum. The spectra of both composites exhibited a shoulder at approx. −85 ppm, which has been shown to represent an amorphous sodium aluminosilicate gel with a molar SiO_2_/Al_2_O_3_ ratio of ∼2 (Si/Al ≈ 1).^26,31^ For CR_3.5_1d, the fraction of Si in that gel has been determined to be 11% of the total Si in the system.^26^

The *S*_0_ spectrum of RHA_3.5_1d exhibited an essentially feature-less, broad hump in the range −75 ppm to −120 ppm, *i.e*. in the full range of Q^4^ and Q^4^(*m*Al) sites with *m* = 1…4. This resembles typical cementitious sodium aluminosilicate gels, which generally contain a distribution of Q^4^(*m*Al) sites with *m* = 1…4, generally with the resonances of the Q^4^(*m*Al) sites broadened so that their ^29^Si single-pulse MAS NMR spectra display only a similar broad hump.^18–23^

**Fig. 2:**
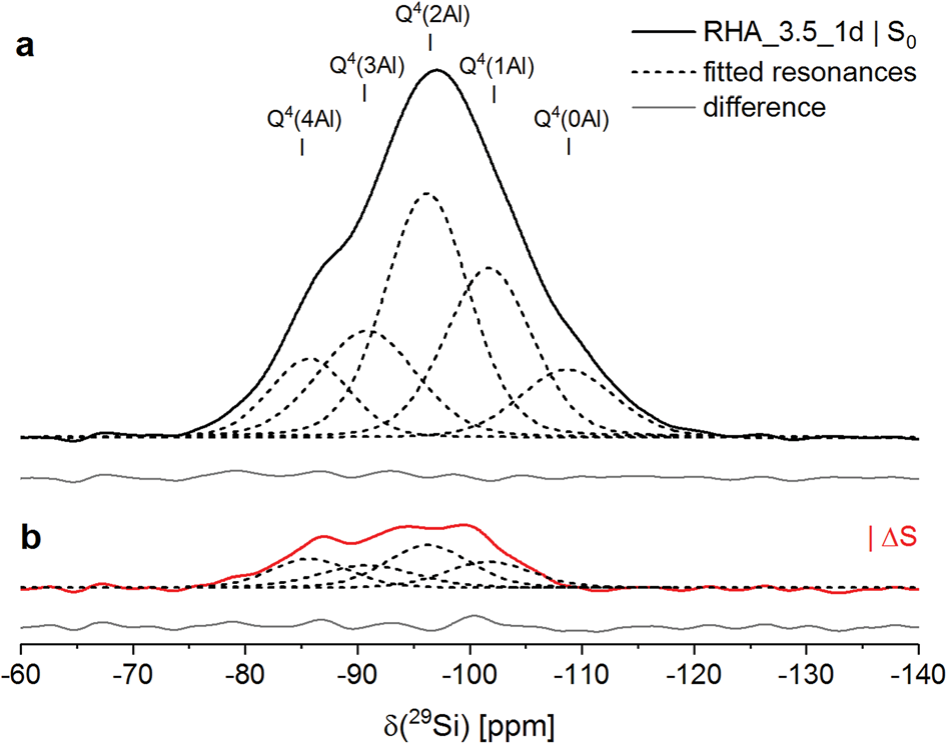
^29^Si{^27^Al} TRAPDOR NMR results for RHA_3.5_1d: experimental *S*_0_ spectrum (a) and difference spectrum Δ*S* = *S*_0_ − *S* (b). In both spectra, fitted resonances are shown as dashed black lines, and the difference between the sum fit and the experimental or difference spectrum is shown as full grey line at the bottom. Experimental conditions as in Fig. 1.

From deconvolution of the ^29^Si{^27^Al} TRAPDOR spectra, the TRAPDOR fractions (Δ*S*/*S*_0_) of the Q^4^(*m*Al) sites for the three materials were obtained (Fig. 2; Table 3). In the composites, the TRAPDOR fractions of the Q^3^ and the Q^4^ species were very low, *viz*. ∼5% for Q^3^ and ∼0% for Q^4^. This is in agreement with the assignment of these sites to the leached surface layer and the inner regions of unreacted silica in the composites, respectively. The slightly raised Δ*S*/*S*_0_ of the Q^3^ sites can be explained by interaction of Si in the surface layer with Al in the surrounding aluminosilicates and with diffusion of a limited amount of Al into the surface layer, leading to interactions with Si residing further inside the hydrated layer. Thus, a well-defined interface with an abrupt change of composition is not present between the unreacted silica and the surrounding zeolites and gel; instead, it appears that there is a more gradual transition between the silica and the aluminosilicate matrix, suggesting that also some bonding exists between these.

**Table 3:** Chemical shifts (*δ*) and TRAPDOR fractions (Δ*S*/*S*_0_) of the Si sites in MS_3.5_1d, CR_3.5_1d and RHA_3.5_1d, obtained from deconvolution of their ^29^Si{^27^Al} TRAPDOR NMR spectra

Material	Phase/site	*δ* (ppm)	Δ*S*/*S*_0_
MS_3.5_1d	Gel Q^4^(4Al)	−84	45%
	Hydrosodalite Q^4^(4Al)	−86	40%
	Zeolite Na-A Q^4^(4Al)	−89	37%
	Silica MS Q^3^	−100	∼5%
	Silica MS Q^4^	−110	∼0%
CR_3.5_1d	Gel Q^4^(4Al)	−85	42%
	Zeolite Na-A Q^4^(4Al)	−89	34%
	Silica CR Q^3^	−100	∼5%
	Silica CR Q^4^	−110	∼0%
RHA_3.5_1d	Gel Q^4^(4Al)	−86	37%
	Gel Q^4^(3Al)	−91	21%
	Gel Q^4^(2Al)	−96	17%
	Gel Q^4^(1Al)	−102	15%
	Silica RHA Q^4^	−109	∼0%

The deconvolution of the ^29^Si{^27^Al} TRAPDOR spectra of RHA_3.5_1d yielded resonances at approx. −86, −91, −96, −102 and −109 ppm, assigned to Q^4^(*m*Al) with *m* = 4, 3, 2, 1 and Q^4^, respectively (Tables 3 and 4). The Q^4^ species are assigned to unreacted silica RHA. Quantification of the relative intensity of that resonance in the ^29^Si single-pulse MAS NMR spectrum of RHA_3.5_1d yielded an abundance of 11% (Table 4). That means that the silica RHA had reacted to a degree of 89% in the material, significantly higher than the degree of reaction of the silicas in MS_3.5_1d and CR_3.5_1d (Section 2.1). As the silica starting materials had comparable specific surface areas, the faster reaction kinetics of RHA are assigned to a higher fraction of network-modifying elements (sum of Na, K, Mg and Ca) in the silica; it is also noted that RHA contained substantial amounts of phosphorus and sulfur, which may increase its reactivity too.

**Table 4:** Relative intensities (*I*) of the Si sites in RHA_3.5_1d, obtained from a fit of its ^29^Si single-pulse MAS NMR spectrum

	Q^4^(4Al)	Q^4^(3Al)	Q^4^(2Al)	Q^4^(1Al)	Q^4^
*δ* (ppm)	−85.6	−90.7	−96.0	−101.6	−108.5
*I*	16%	19%	35%	19%	11%

The Q^4^(*m*Al) sites with *m* = 1…4 in RHA_3.5_1d represent the sodium aluminosilicate gel (geopolymeric gel),^20,21,29,40^*i.e*. the product of the reaction of the silica RHA with the sodium aluminate. From their relative intensities (*I*) in the ^29^Si single-pulse MAS NMR spectrum, the molar SiO_2_/Al_2_O_3_ ratio of the sodium aluminosilicate gel, excluding the unreacted silica RHA, can be computed with Engelhardt's formula,^41^1
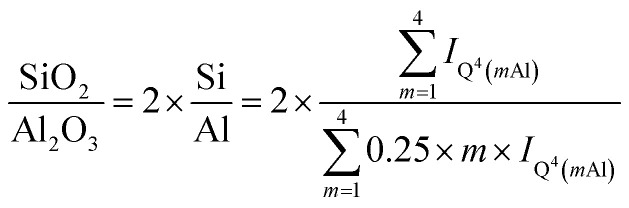
provided that virtually no Al–O–Al bonds are present in the material (Loewenstein's rule). The latter assumption has been confirmed by ^17^O 3QMAS NMR for cementitious sodium aluminosilicate gels with Si/Al > 1, produced by alkali-activation of synthetic precursors.^28^ Insertion of the intensities of Q^4^(*m*Al) with *m* = 1…4, shown in Table 4, into eqn (1) yields SiO_2_/Al_2_O_3_ = 3.78 (Si/Al = 1.89) for the sodium aluminosilicate gel. This value is higher than the starting SiO_2_/Al_2_O_3_ ratio of RHA_3.5_1d (SiO_2_/Al_2_O_3_ = 3.48; Table 2), showing that some of the Al from the sodium aluminate had not entered the sodium aluminosilicate gel.

The TRAPDOR fractions of the Q^4^(*m*Al) sites in the sodium aluminosilicate gel in MS_3.5_1d, CR_3.5_1d, RHA_3.5_1d, zeolite Na-A and the hydrosodalite were in relative good agreement (Table 3), considering that their determination involved separate deconvolution for each of the materials. However, the slightly lower Δ*S*/*S*_0_ of Q^4^(4Al) in the gel of RHA_3.5_1d, compared to the Q^4^(4Al) in the gels of the composites, may be partly caused by its higher overall SiO_2_/Al_2_O_3_ ratio, as the TRAPDOR fraction is not only determined by next-nearest neighbor Al atoms (Si–O–Al bonds) but also by Al that resides further away from the nucleus under study (*e.g.*, Si–O–Si–O–Al).

The TRAPDOR fractions of the Q^4^(*m*Al) sites in the gel of RHA_3.5_1d decreased with decreasing *m* in the expected order (Table 3). Δ*S*/*S*_0_ of the Q^4^(1Al) sites in RHA_3.5_1d was determined to be 15%, *i.e*. considerably higher than that of the Q^3^ sites in the composites but only slightly lower than the TRAPDOR fraction of the Q^4^(2Al) sites in RHA_3.5_1d. This indicates that any Q^3^ species in a leached surface layer of the unreacted silica RHA, if present at all, contributed only to a minor extent to the resonance assigned to Q^4^(1Al). This would also be in line with the high degree of reaction of RHA. Because of this, the above use of the intensity of the Q^4^(1Al) sites, without a reduction to account for possible Q^3^ species, to calculate the SiO_2_/Al_2_O_3_ ratio of the aluminosilicate gel is justified.

### 3.2 ^27^Al{^29^Si} and ^27^Al{^1^H} REDOR NMR

**Fig. 3:**
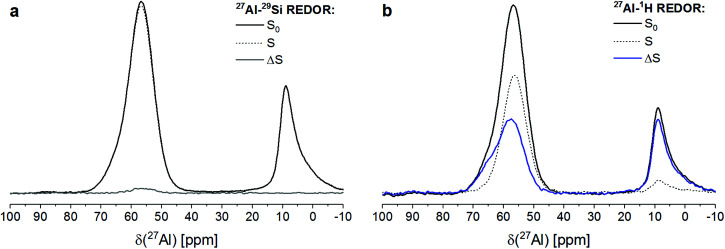
^27^Al{^29^Si} (a) and ^27^Al{^1^H} (b) REDOR NMR spectra of RHA_3.5_1d. *S*_0_ (full black lines): spectrum obtained without REDOR pulse; *S* (dashed black lines): spectrum obtained for an echo time of 2.40 ms (*N* = 30); Δ*S* (full grey or blue lines): difference spectrum Δ*S* = *S*_0_ − *S*.

RHA_3.5_1d contained significant amounts of aluminium in four-fold coordination (AlO_4_) as well as in six-fold coordination (AlO_6_), as shown by its ^27^Al REDOR NMR *S*_0_ spectra (Fig. 3). The maximum of the six-coordinated Al was located at a chemical shift of 8 ppm. The four-coordinated Al caused a resonance with its maximum at 58 ppm and a shoulder, centered at ∼65 ppm. As the resonance of Al in sodium aluminate in ^27^Al MAS NMR spectra is found at ∼77–78 ppm,^42,43^ this proves that the sodium aluminate has dissolved and reacted virtually completely during curing of RHA_3.5_1d.

The signal of AlO_6_ did not exhibit a discernable ^27^Al{^29^Si} REDOR NMR effect (Fig. 3a), while its ^27^Al{^1^H} REDOR NMR effect was determined to be 88% (Fig. 3b). This proves that the six-coordinated Al existed in a proton-rich (*i.e*. water-rich) environment with no or only little Si in proximity, strongly suggesting that it formed a hydrous aluminate phase. The occurrence of a separate aluminate phase in RHA_3.5_1d is in line with the above finding that the SiO_2_/Al_2_O_3_ ratio of its sodium aluminate gel was higher than the overall SiO_2_/Al_2_O_3_ ration of the starting mix and the complete reaction of the sodium aluminate. As no crystalline compounds were detected by XRD in RHA_3.5_1d (Section 2.1), this must be an amorphous aluminate, *i.e*. alumina gel. This interpretation is in accord with work of Brew and MacKenzie,^43^ who detected minor amounts of AlO_6_ in samples produced by addition of silica fume to sodium aluminate solution and assigned these to poorly ordered Al(OH)_3_. The same reasoning has been adopted previously to explain the occurrence of minor amounts of AlO_6_ in MS_3.5_1d and related materials with other SiO_2_/Al_2_O_3_ ratios.^31^ The ^27^Al{^29^Si} and ^27^Al{^1^H} REDOR NMR results presented here confirm these previous assignments.

The two AlO_4_ species at 58 ppm and ∼65 ppm, respectively, displayed different behavior regarding their coupling with Si and protons (Fig. 3). The major signal at 58 ppm exhibited a ^27^Al{^1^H} REDOR NMR effect of 39%, *i.e*. lower than the effect of the AlO_6_ sites, and a ^27^Al{^29^Si} REDOR NMR effect of 2.5%. The latter figure has to be considered as indicating abundant Si in spatial proximity, as only ^29^Si isotopes (natural isotope abundance 4.7%) contribute to Δ*S*/*S*_0_. The shoulder at ∼65 ppm exhibited a ^27^Al{^1^H} REDOR NMR effect similar to that of the AlO_6_ sites, indicating a water-rich environment, while a significant ^27^Al{^29^Si} REDOR NMR effect was not observed.

**Fig. 4:**
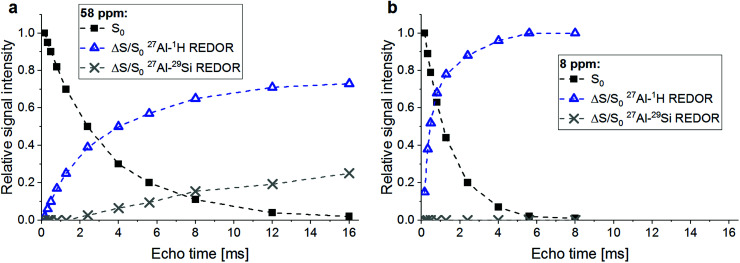
^27^Al{^29^Si} and ^27^Al{^1^H} REDOR effects (Δ*S*/*S*_0_) and relative intensities (*S*_0_) for the AlO_4_ resonance at 58 ppm (a) and the AlO_6_ resonance at 8 ppm (b) of RHA_3.5_1d *versus* echo time. Experimental conditions as in Fig. 3.

A confirmation of the observations regarding the AlO_4_ resonance at 58 ppm and the AlO_6_ resonance at 8 ppm is obtained from a plot of the Δ*S*/*S*_0_*versus* echo time (Fig. 4). Already the faster decay with increasing echo times of the *S*_0_ signal of the AlO_6_ resonance, compared to the AlO_4_ resonance, indicates shorter spin–spin relaxation times (*T*_2_), which results from a higher water abundance around the former. This is confirmed by its much steeper increase of its ^27^Al{^1^H} REDOR NMR effect with echo time. Also, the maximum ^27^Al{^1^H} REDOR NMR effect of the AlO_6_ resonance (∼100%) was significantly higher than that of the AlO_4_ resonance (73% at 2*τ* = 16.00 ms). The ^27^Al{^29^Si} REDOR effect of the AlO_4_ resonance increased approximately linear with echo time to 25% at 16.00 ms, while no Al–Si coupling could be detected for the AlO_6_ resonance, even at long echo times.

From the above results, it is clear that the four-coordinated Al with its resonance at 58 ppm is Al in the framework of the sodium aluminosilicate gel, in line with previous ^27^Al MAS NMR studies of these materials.^18,19,22,23,31,40^ It has Si in close proximity (Al–O–Si bonds), and the abundance of water in its proximity is significant (water of hydration of charge balancing ions and pore water in the gel) but less than for the Al in the alumina gel.

The AlO_4_ sites with their resonance at ∼65 ppm, which have few or no close Si atoms (Al–O–Si bonds) but abundant water in proximity, could be thought to be four-coordinated extra-framework Al (EFAL) species. The existence of four-coordinated EFAL in cementitious aluminosilicate gels has been proposed by Brus *et al.*,^25^ referring to the occurrence of such species in dealuminated zeolite H–Y.^44,45^ The authors gave the ^27^Al isotropic chemical shifts (*δ*_iso_) of the framework Al and the EFAL in their aluminosilicate gel as ∼61 ppm and ∼69 ppm, respectively, reasonably close to the experimental chemical shifts (*δ*) of the two AlO_4_ species in the present study. However, it may be noted that four-coordinated EFAL in dealuminated zeolite H–Y is not always stable in the presence of excess water: while it persists up to at least 80% relative humidity in steamed zeolite H–Y,^44^ it transforms to six-coordinated EFAL at high water loadings in calcined zeolite H–Y.^46^ An alternative explanation for the occurrence of two different resonances in the AlO_4_ range of the ^27^Al MAS NMR spectra of sodium aluminosilicate gels has been proposed recently.^28^ In that study, resonances were found at isotropic chemical shifts of ∼61 ppm and ∼66 ppm and assigned to framework Al balanced by Na^+^ and framework Al balanced by six-coordinated EFAL, respectively. However, adopting this assignment for the two AlO_4_ species in the present study would require that the species causing the signal at ∼65 ppm had abundant Si in proximity (*i.e*. framework Al), which is not the case here. Therefore, this explanation can be excluded for the present materials. Another possibility is that the signal at ∼65 ppm in RHA_3.5_1d is caused by a minor amount of AlO_4_ sites in its hydrous alumina gel. For example, Isobe *et al.*^47^ have identified AlO_4_ with a ^27^Al chemical shift of 65–68 ppm as well as AlO_5_ species besides AlO_6_ in amorphous aluminium hydroxide precipitated from AlCl_3_/NaOH solution, proving that not necessarily all Al in alumina gel is in six-fold coordination. From the present results, it cannot be conclusively decided whether this latter explanation (AlO_4_ in alumina gel) or the possible explanation mentioned first (four-coordinated EFAL) applies to the sites with a resonance at ∼65 ppm in RHA_3.5_1d, but the above reasoning about the stability of four-coordinated EFAL in the presence of excess water may be taken as an indication in favor of AlO_4_ as a minor constituent of the alumina gel.

## 4 Conclusions

^29^Si{^27^Al} TRAPDOR NMR, ^27^Al{^29^Si} and ^27^Al{^1^H} REDOR NMR experiments provide information about alkali-activated materials that is otherwise difficult to obtain and thus are useful methods to complement single-pulse MAS NMR studies. In particular, they allow to unequivocally identify amorphous byproducts such as amorphous alumina gel and they contribute to a reliable identification, and thus quantification, of unreacted amorphous precursors in these materials.

As for the materials studied here, ^29^Si{^27^Al} TRAPDOR NMR experiments have confirmed that the composites based on silica MS and silica CR contained substantial amounts of unreacted silica with a leached surface layer. ^27^Al{^29^Si} and ^27^Al{^1^H} REDOR NMR experiments revealed that the amorphous material based on silica RHA was not a phase-pure sodium aluminosilicate gel but contained coprecipitated hydrous alumina gel. The RHA-based material contained unreacted silica in addition, but its amount was markedly smaller than in the composites. The combination of the latter two methods also showed that two four-coordinated Al species occurred in the RHA-based material, one of which was the framework Al in the aluminosilicate gel, while the other was likely AlO_4_ in the alumina gel or possibly four-coordinated extra-framework Al in the aluminosilicate gel.

The results demonstrate that the choice of the silica feedstock in the production of one-part alkali-activated materials has a very important effect on the phase assemblage of the cured products: though all employed silicas were amorphous, the materials synthesized from MS and CR were gel–zeolite composites containing a substantial amount of unreacted silica (*i.e*. ‘excess’ silica), while the materials synthesized from the rice husk ash RHA was a completely amorphous sodium aluminosilicate gel containing hydrous alumina gel as byproduct (*i.e*. ‘excess’ alumina). It is noted that occurrence of alumina gel is likely an advantage for the previously proposed application of one-part alkali-activated materials as binders for sewer repair mortars.^5^ One of the most important aspects for this application is the resistance of the mortars against biogenic sulfuric acid attack. It has been proposed^48,49^ that Al(OH)_3_ in calcium aluminate cements increases the resistance against biogenic sulfuric acid attack of mortars and concretes produced from these cements by releasing bacteriostatic Al^3+^ into solution and thus inhibiting the activity of sulfuric acid-producing bacteria on the surface of the mortar or concrete. This suggests that coprecipitated hydrous alumina gel in one-part alkali-activated materials, which was observed here for RHA_3.5_1d, would have the same effect.

The different phase assemblages of the materials based on the different silica starting materials can likely be explained by differences of the dissolution kinetics of the silicas under alkaline conditions, caused by differences of the amounts of network-modifying constituents. This suggests that choice of a silica starting material with suitable dissolution kinetics, adjusted to the overall SiO_2_/Al_2_O_3_ ratio of the reaction mixture, will lead to virtually complete conversion of the starting materials into phase-pure sodium aluminosilicate gel, which can impart improved engineering properties, *e.g.*, higher mechanical strength, to the material. It may also be possible to yield the same effect by mixing different silica starting materials, but this suggestion has to tested in future studies. If true, this would add another benefit to one-part alkali-activated materials, which, due to the avoidance of the employment of highly alkaline activator solutions, have already substantial advantages in terms of safety and handling compared to alkali-activated materials produced by the conventional synthesis route.^30,50–52^

## Conflicts of interest

The authors declare no competing interests.

## Supplementary Material
