# Correction: ^29^Si{^27^Al}, ^27^Al{^29^Si} and ^27^Al{^1^H} double-resonance NMR spectroscopy study of cementitious sodium aluminosilicate gels (geopolymers) and gel–zeolite composites

**DOI:** 10.1039/d3ra90078a

**Published:** 2023-09-01

**Authors:** Sebastian Greiser, Gregor J. G. Gluth, Patrick Sturm, Christian Jäger

**Affiliations:** a Division 1.3 Structure Analysis, Bundesanstalt für Materialforschung und-prüfung (BAM) Richard-Willstätter-Str. 11 12489 Berlin Germany christian.jaeger@bam.de; b Division 7.4 Technology of Construction Materials, Bundesanstalt für Materialforschung und-prüfung (BAM) Unter den Eichen 87 12205 Berlin Germany gregor.gluth@bam.de

## Abstract

Correction for ‘^29^Si{^27^Al}, ^27^Al{^29^Si} and ^27^Al{^1^H} double-resonance NMR spectroscopy study of cementitious sodium aluminosilicate gels (geopolymers) and gel–zeolite composites’ by Sebastian Greiser *et al.*, *RSC Adv.*, 2018, **8**, 40164–40171, https://doi.org/10.1039/C8RA09246J.

The authors regret that there was an arithmetical error in the calculation of the molar SiO_2_/Al_2_O_3_ ratio of the sodium aluminosilicate gel in RHA_3.5_1d in the original article. The correct value is 3.39. The corresponding text [following eqn (1) in Section 3.1] should thus read:

“Insertion of the intensities of Q^4^ (*m*Al) with *m* = 1…4, shown in Table 4, into eqn (1) yields SiO_2_/Al_2_O_3_ = 3.39 (Si/Al = 1.70) for the sodium aluminosilicate gel. This value is very close to the starting SiO_2_/Al_2_O_3_ ratio of RHA_3.5_1d (SiO_2_/Al_2_O_3_ = 3.48; Table 2); together with the fact that the silica RHA had reacted only incompletely (degree of reaction: 89%) this shows that some of the Al from the sodium aluminate had not entered the sodium aluminosilicate gel.”

This change requires an additional minor change of a statement in the second paragraph of Section 3.2, which should read:

“The occurrence of a separate aluminate phase in RHA_3.5_1d is in line with the above finding that the SiO_2_/Al_2_O_3_ ratio of its sodium aluminate gel was very similar to the overall SiO_2_/Al_2_O_3_ ratio of the starting mix and the complete reaction of the sodium aluminate, while the silica RHA had reacted only incompletely.”

An independent expert has viewed the corrected data and has concluded that it is consistent with the discussions and conclusions presented.

The Royal Society of Chemistry apologises for these errors and any consequent inconvenience to authors and readers.

## Supplementary Material

